# Modelo teórico de promoção da alimentação adequada e saudável na Educação Infantil

**DOI:** 10.1590/0102-311XPT213524

**Published:** 2025-08-08

**Authors:** Rafaele Febrone, Inês Rugani Ribeiro de Castro, Rosane Valéria Viana Fonseca Rito

**Affiliations:** 1 Instituto de Nutrição, Universidade do Estado do Rio de Janeiro, Rio de Janeiro, Brasil.; 2 Faculdade de Nutrição, Universidade Federal Fluminense, Niterói, Brasil.

**Keywords:** Criança, Creches, Aleitamento Materno, Alimentação Complementar, Educação Alimentar e Nutricional, Child, Child Day Care Centers, Breast Feeding, Complementary Feeding, Food and Nutrition Education, Niño, Guarderías Infantiles, Lactancia Materna, Alimentación Complementaria, Educación Alimentaria y Nutricional

## Abstract

Este estudo teórico-conceitual objetivou desenvolver modelo teórico de promoção da alimentação adequada e saudável na Educação Infantil, por meio de: levantamento bibliográfico, elaboração e validação de face e de conteúdo do modelo. Foram adotados como referenciais teórico-conceituais as vertentes da promoção da alimentação adequada e saudável e a Teoria da Estruturação Forte. O modelo proposto consiste em um esquema visual que apresenta ações práticas de promoção a serem desenvolvidas por agentes do contexto interno e externo à unidade. Seu desenvolvimento pressupõe articulação intersetorial e abrange ações de: incentivo à adoção de hábitos alimentares saudáveis (inclusão da educação alimentar e nutricional como prática cotidiana e transversal ao currículo, mobilização e formação sobre este assunto junto às famílias); apoio à adesão de práticas alimentares saudáveis (oferta de leite humano e de alimentação adequada e saudável, atendimento a crianças com necessidades alimentares especiais ou específicas); proteção à alimentação adequada e saudável (controle higiênico-sanitário da alimentação, prevenção de acidentes nos ambientes de preparo e de oferta de refeições, regulação de oferta, comércio e publicidade de alimentos ultraprocessados e de preparações não recomendadas), complementadas pelas ações transversais (formação, interação, monitoramento e avaliação). O modelo proposto contribui para a compreensão sobre o tema ao explicitar o escopo de ações da unidade de Educação Infantil concernente à promoção da alimentação adequada e saudável.

## Abstract (en)

This theoretical-conceptual study aimed to develop a theoretical model to promote adequate and healthy eating in early childhood education by surveying the literature, developing a model, and evaluating its face and content validity. The axes of promoting adequate and healthy eating and the Strong Structuring Theory were adopted as theoretical-conceptual references. The proposed model consists of a visual scheme that shows actions to be developed by agents inside and outside early childhood education units. Its development presupposes intersectoral articulations and actions to encourage the adoption of healthy eating habits (inclusion of food and nutrition education as a daily and transversal practice to the curriculum, and mobilization and training on this topic with families), support for adherence to healthy eating practices (supply of human milk and adequate and healthy food and care for children with special or specific dietary needs), and protect healthy and adequate food (control the hygiene of food; accident prevention in the environments in which meals are prepared and offered; and regulation of the supply, trade, and advertising of ultra-processed foods and non-recommended preparations) along with transversal actions (training, interaction, monitoring, and evaluation). The proposed model contributes to the understanding of the topic by explaining the scope of early childhood education unit actions concerning the promotion of adequate and healthy eating.

## Resumen

Este estudio teórico-conceptual tuvo como objetivo desarrollar un modelo teórico para promover una alimentación adecuada y saludable en la educación infantil a partir de: encuesta bibliográfica; desarrollo y validación aparente y de contenido del modelo. Se utilizaron como marcos teórico-conceptuales los ejes de la promoción de una alimentación adecuada y saludable y de la Teoría de Estructuración Fuerte. El modelo propuesto consiste en un esquema visual que presenta acciones de promoción que serán desarrolladas por agentes del contexto interno y externo de la unidad de educación infantil. Su desarrollo presupone articulación intersectorial e incluye acciones para: fomentar la adopción de hábitos alimentarios saludables (inclusión de la educación alimentaria y nutricional como práctica diaria y transversal al currículo y movilización y formación sobre este tema con las familias); apoyo a la adhesión a prácticas alimentarias saludables (suministro de leche humana y alimentación adecuada y saludable y atención a niños con necesidades dietéticas especiales o específicas); protección a alimentación adecuada y saludable (control higiénico-sanitario de los alimentos, prevención de accidentes en entornos de preparación y provisión de comidas; y regulación del suministro, comercio y publicidad de alimentos ultraprocesados y preparados no recomendados), complementado con acciones transversales (formación, interacción, seguimiento y evaluación). El modelo propuesto contribuye a la comprensión sobre el tema al hacer explícito el alcance de las acciones de la unidad de educación infantil con respecto a la promoción de una alimentación adecuada y saludable.

## Introdução

A promoção da alimentação adequada e saudável é prioridade na agenda de políticas públicas de alimentação e nutrição ^1^ ^,^ ^2^ e uma das diretrizes do Programa Nacional de Alimentação Escolar (PNAE) no Brasil ^3^. Ela consiste em um conjunto de estratégias que proporcionam aos indivíduos e coletividades a realização de práticas alimentares apropriadas aos seus aspectos biológicos e socioculturais, em acordo com as necessidades de cada fase do curso da vida e com as necessidades alimentares especiais, bem como com o uso sustentável dos recursos naturais ^1^. A promoção da alimentação adequada e saudável na primeira infância é um dever de responsabilidade setorial e intersetorial sinérgica, compartilhado pelas famílias, sociedade e pelo Estado, e uma prioridade global ^4^.

As unidades de Educação Infantil têm papel determinante no crescimento e no desenvolvimento integral, na promoção da saúde, da alimentação adequada e saudável e do autocuidado das crianças de até cinco anos, especialmente por contribuírem para a formação cidadã, de valores e de hábitos de vida. São, portanto, ambiente propício para o desenvolvimento de estratégias de promoção da alimentação adequada e saudável que envolvam toda a comunidade escolar ^3^ ^,^ ^5^ ^,^ ^6^.

Nessa perspectiva, as unidades de Educação Infantil devem garantir: a possibilidade de interação de seus agentes para o enfrentamento dos desafios cotidianos em relação ao processo de desenvolvimento das crianças; a coerência entre a rotina escolar que envolve a alimentação, as práticas educativas e as abordagens de alimentação adequada e saudável presentes nos documentos orientadores de políticas públicas; e o desenvolvimento de ações alinhadas às três vertentes da promoção da alimentação adequada e saudável: incentivo; apoio, e proteção; complementadas por ações transversais ^7^ ^,^ ^8^ ^,^ ^9^.

Ainda é incipiente na literatura o desenvolvimento de arcabouço teórico que fundamente estratégias de promoção da alimentação adequada e saudável condizentes com o contexto da Educação Infantil, e que integrem as práticas alimentares saudáveis com as atividades pedagógicas desenvolvidas nessas instituições. Buscando contribuir para a superação dessa lacuna, este estudo objetivou desenvolver modelo teórico de promoção da alimentação adequada e saudável na Educação Infantil com vistas a facilitar a compreensão desse tema e apoiar a qualificação e melhoria das práticas de promoção da alimentação adequada e saudável no âmbito da Educação Infantil. O modelo teórico proposto expressa a realidade brasileira, especificamente das unidades de Educação Infantil públicas, mas é aplicável a outras realidades semelhantes.

## Método

Trata-se de um estudo teórico-conceitual que contemplou as seguintes etapas: (1) levantamento bibliográfico nos meios eletrônicos, buscando-se publicações e documentos orientadores de políticas públicas oriundos de organizações governamentais, agências e entidades da sociedade civil nacionais e internacionais, além de normativas e marcos legais que abordam a promoção da alimentação adequada e saudável no ambiente escolar e temas afins, como alimentação escolar, ambiente alimentar escolar, educação alimentar e nutricional voltada à primeira infância, prática da alimentação adequada e saudável em unidades de Educação Infantil e proteção à infância, e buscas ativas durante a leitura dos documentos encontrados ^10^; (2) sistematização e síntese das ações de promoção da alimentação adequada e saudável em unidades de Educação Infantil recomendadas na bibliografia levantada; e (3) desenvolvimento do modelo teórico propriamente dito (esquema gráfico e seus quadros explicativos), que incluiu sua elaboração e a avaliação de sua validade de face e de conteúdo.

O modelo teórico é entendido como um esquema visual de uma teoria, com objetivo de expressar as relações existentes entre os conceitos, de forma a compreender uma realidade. Sua construção é recomendada com o objetivo de organizar conceitos de uma teoria, fundamentar a construção de instrumentos de aferição, orientar ações de um dado programa ou política pública e/ou estudos e intervenções voltados para determinado tema ^11^.

### Bases teóricas e conceituais para construção do modelo proposto

Foi adotada como um dos referenciais teórico-conceituais deste estudo a concepção de que a promoção da alimentação adequada e saudável engloba três vertentes de ação: incentivo, apoio e proteção ^5^ ^,^ ^7^ ^,^ ^8^ ^,^ ^9^. A vertente de incentivo abarca, fundamentalmente, ações de difusão de informação e de motivação para a adoção de hábitos alimentares saudáveis; a de apoio engloba todas as medidas que visam a tornar factíveis e/ou a facilitar a adesão a práticas alimentares saudáveis por pessoas informadas e motivadas; e a de proteção à alimentação adequada e saudável abrange iniciativas voltadas a evitar práticas alimentares inadequadas ^8^ ^,^ ^9^ ^,^ ^7^. Esse referencial foi utilizado para orientar a sistematização da literatura sobre o tema e para desenvolver elementos centrais do modelo teórico referentes à promoção da alimentação adequada e saudável propriamente dita.

O segundo referencial teórico adotado para a estruturação do modelo foi a Teoria da Estruturação Forte (TEF). Ela se aplica a estudos voltados à compreensão de processos e contextos organizacionais, ao permitir a análise de objetos de pesquisa constituídos por mais de um agente em foco. Essa teoria apresenta o conceito da dualidade da estrutura como quatro componentes passíveis de serem analisados separadamente, mas interligados de forma cíclica, denominados de ciclo quadripartido da estruturação, a saber: estruturas externas ao agente em foco (condições de ação dentro da estruturação); estruturas internas do agente (conhecimento conjunturalmente específico e disposições gerais); agência ativa ou práticas dos agentes (maneira pela qual um agente em foco age com base nas suas estruturas internas); e resultados da agência ativa (efeitos das práticas dos agentes em foco que afetam ambas as estruturas) ^12^.

No presente estudo, o contexto organizacional de interesse se refere ao âmbito da unidade de Educação Infantil, entendendo que a institucionalização, concretização, longevidade e continuidade das ações de promoção da alimentação adequada e saudável pressupõe uma organização institucional. A TEF foi usada para conceber o modelo teórico, estabelecer alguns conceitos e identificar os possíveis agentes e os contextos interno e externo da unidade de Educação Infantil. Essa teoria também foi adotada tendo em vista que, por meio do ciclo quadripartido da estruturação, ela articula elementos que possibilitam a compreensão de como se estabelece a organização da promoção da alimentação adequada e saudável no contexto da Educação Infantil.

### Avaliação da validade de face e de conteúdo do modelo proposto

Com base no levantamento bibliográfico e nos referenciais teórico-conceituais adotados, uma primeira versão do modelo foi elaborada pelas autoras. Sua validade de face foi avaliada por meio de painel de especialistas. Foram realizadas duas oficinas virtuais, uma em dezembro de 2022 (n = 13) e outra em maio de 2023 (n = 10), com os objetivos de analisar e aprimorar as duas primeiras versões do modelo teórico. As versões preliminares do modelo foram enviadas previamente às especialistas e apresentadas durante as oficinas. Em discussões em grupos e em plenária, as especialistas apresentaram suas impressões gerais sobre o conteúdo e a representação gráfica do modelo, e indicaram conteúdos e elementos a serem incluídos, excluídos ou editados/detalhados. Após cada oficina, houve o aprimoramento do modelo com base nas sugestões das especialistas.

A validade de conteúdo foi avaliada, entre maio e junho de 2023, por meio de procedimentos qualitativos (comentários) e quantitativos (taxa de validade de conteúdo, em inglês *content validity ratio* [CVR]) ^11^. Cada especialista (n = 10) respondeu assincronamente um formulário eletrônico com uma lista dos aspectos do modelo teórico a serem avaliados em uma escala de quatro pontos, considerando: 4 = “altamente relevante”; 3 = “bastante relevante” ou “altamente relevante, mas precisa ser reescrita”; 2 = “pouco relevante”; e 1 = “não relevante”.

Os aspectos avaliados nessa etapa foram organizados em três módulos, referentes aos elementos constitutivos do modelo: (1) esquema gráfico (a disposição das vertentes de promoção da alimentação adequada e saudável, dos 12 componentes do modelo, dos contextos interno e externo à unidade de Educação Infantil, de seus agentes, e as cores usadas); (2) quadro de descrição dos elementos estruturantes do modelo (a disposição e o detalhamento dos contextos interno e externo à unidade de Educação Infantil; dos agentes do contexto interno e externo à unidade de Educação Infantil; e das vertentes de promoção da alimentação adequada e saudável), e (3) quadro de definições e exemplos dos componentes do modelo (as definições dos componentes; os exemplos de práticas, e sugestões de agentes envolvidos nas práticas dos 12 componentes do modelo). Cada aspecto foi analisado e avaliado individualmente.

O CVR de cada aspecto foi obtido pela aplicação da seguinte fórmula: CVR = (n_e_ - N/2)/(N/2), em que “n_e_” é o número de especialistas que classificaram cada aspecto como “essencial” (4 ou 3) e “N” é o número total de respondentes. O CVR varia entre -1 e +1, e o valor 0 significa que metade do painel considera o aspecto essencial ^11^ ^,^ ^13^. Considerando que dez especialistas participaram nessa etapa do estudo, buscando-se evitar que o resultado fosse devido ao acaso, foi adotado o ponto de corte de 0,62, proposto por Lawshe ^14^ para se considerar um aspecto essencial. Aspectos com valores abaixo desse seriam descartados.

No formulário, havia espaço para comentários sobre cada aspecto do modelo que o avaliador considerasse que deveria ser incluído, excluído ou editado, e perguntas referentes à clareza do esquema gráfico e de seus quadros explicativos. Após essa etapa, foram sistematizados os resultados e realizados os ajustes finais no modelo teórico. O aprimoramento do esquema gráfico contou com o trabalho de uma designer profissional, realizado sob orientação da equipe de pesquisa.

O estudo contou com a participação de 14 especialistas ligadas à agenda de alimentação e nutrição, com as seguintes expertises: alimentação escolar (n = 6); ambiente alimentar e saúde (n = 2); saúde coletiva (n = 3); segurança alimentar e nutricional (n = 1); desenvolvimento de modelos teóricos (n = 1); e gestão pública (n = 1).

### Aspectos éticos

A pesquisa foi aprovada pelo Comitê de Ética em Pesquisa da Universidade do Estado do Rio de Janeiro (UERJ; parecer nº 5.811.823/2022 e CAAE nº 62602822.5.0000.5259).

## Resultados

### Modelo teórico de promoção da alimentação adequada e saudável na Educação Infantil

O modelo teórico de promoção da alimentação adequada e saudável na Educação Infantil consiste em um esquema visual que apresenta as ações de promoção da alimentação adequada e saudável a serem desenvolvidas no âmbito da unidade de Educação Infantil, considerando seus contextos interno e externo. Seu desenvolvimento compreende o planejamento e a execução articulada de ações que se interconectam, de responsabilidade compartilhada por agentes do contexto interno e externo à unidade de Educação Infantil, de: incentivo à adoção de hábitos alimentares saudáveis, por meio da incorporação da educação alimentar e nutricional como prática cotidiana e transversal ao currículo, e da mobilização e formação sobre a promoção da alimentação adequada e saudável junto às famílias; apoio à adesão de práticas alimentares saudáveis, por meio da oferta de leite humano e de alimentação adequada e saudável, e do atendimento a crianças com necessidades alimentares especiais ou específicas; proteção à alimentação adequada e saudável, por meio de medidas de controle higiênico-sanitário da alimentação, prevenção de acidentes nos ambientes de preparo e oferta de refeições e regulação da oferta, do comércio e da publicidade de alimentos ultraprocessados e preparações não recomendadas; complementadas pelas ações transversais de formação dos profissionais, de interação com outros agentes, setores ou instâncias e de monitoramento e avaliação.

**Figura 1:**
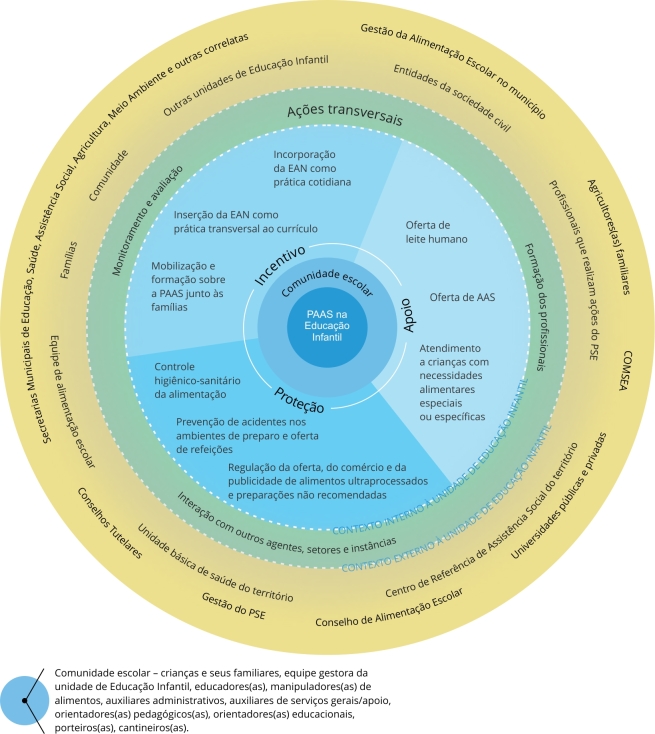
Esquema gráfico do modelo teórico de promoção da alimentação adequada e saudável na Educação Infantil.

Os elementos constitutivos do modelo teórico são o seu esquema gráfico (Figura 1) e seus quadros explicativos: o de descrição dos elementos estruturantes do modelo (Quadro 1) e o de definições e exemplos dos componentes do modelo (Quadro 2).

**Quadro 1:** Descrição dos elementos estruturantes do modelo teórico de promoção da alimentação adequada e saudável na Educação Infantil.

ELEMENTO	DETALHAMENTO
CONTEXTO	
Externo à unidade de Educação Infantil	Abarca as condições do território; o tecido social; o nível de articulação que os diferentes agentes, instituições, organizações, setores e instâncias que interagem com a unidade de Educação Infantil estabelecem e mantêm. Circunscreve-se ao âmbito do município e pode ser dividido em: (a) proximal, que se refere ao contexto que envolve os agentes, setores e instâncias presentes no território em que a unidade de Educação Infantil está inserida e suas interações, incluindo as características do ambiente alimentar do território; e (b) distal, que se refere ao contexto que envolve agentes, setores e instâncias que atuam em nível municipal e suas interações com a unidade de Educação Infantil, menos cotidianas e diretas que aquelas do nível proximal
Interno à unidade de Educação Infantil	Espaço físico (infraestrutura) e contexto institucional (correlações de forças, cultura institucional e todos os elementos relacionados com a organização, e todas as práticas e processos referentes à educação, aí incluídos os elementos da alimentação e nutrição) da unidade de Educação Infantil, incluindo as características do ambiente alimentar escolar
AGENTES, SETORES E INSTÂNCIAS	
Do contexto externo à unidade de Educação Infantil	Agentes, instituições, organizações, setores e instâncias externos à unidade de Educação Infantil, que atuam em âmbito municipal, que podem atuar direta ou indiretamente junto ao processo de educação das crianças, divididos em proximal e distal. Contexto externo proximal: famílias das crianças atendidas na unidade de Educação Infantil, comunidade, outras unidade de Educação Infantil, equipe de alimentação escolar, unidade básica de saúde do território, Centro de Referência de Assistência Social do território, profissionais que realizem ações do Programa Saúde na Escola, entidades da sociedade civil. Contexto externo distal: Secretarias Municipais de Educação, Saúde, Assistência Social, Agricultura, Meio Ambiente e outras correlatas, Gestão de Alimentação Escolar no município, agricultores(as) familiares, Conselho Municipal de Segurança Alimentar e Nutricional, Conselho de Alimentação Escolar, Conselhos Tutelares, gestão do Programa Saúde na Escola, universidades públicas e privadas (incluindo os Centros Colaboradores em Alimentação Escolar - CECANE)
Do contexto interno à unidade de Educação Infantil	Agentes internos à unidade de Educação Infantil que atuam diretamente junto ao processo de educação das crianças, constituindo a comunidade escolar. São eles: crianças e seus familiares, equipe gestora da unidade de Educação Infantil, educadores(as), manipuladores(as) de alimentos, auxiliares administrativos, auxiliares de serviços gerais/apoio, orientadores(as) pedagógicos(as), orientadores(as) educacionais, porteiros(as) e cantineiros(as)
VERTENTES DA PROMOÇÃO DA ALIMENTAÇÃO ADEQUADA E SAUDÁVEL	
Incentivo	Ações que visam informar e motivar os indivíduos à adoção de práticas alimentares saudáveis, por meio de atividades de educação alimentar e nutricional junto à comunidade escolar - crianças e seus familiares, equipe gestora da unidade de Educação Infantil, educadores(as), manipuladores(as) de alimentos, auxiliares administrativos, auxiliares de serviços gerais/apoio, orientadores(as) pedagógicos(as), orientadores(as) educacionais, porteiros(as), cantineiros(as) - que informem, promovam a reflexão e motivem escolhas saudáveis
Apoio	Medidas que visam facilitar a adesão a práticas alimentares saudáveis, por meio da garantia da oferta de leite humano e de alimentos adequados e saudáveis às crianças, respeitando-se a fase da vida, as necessidades individuais e a cultura alimentar, garantindo-se a adequação das condições higiênico-sanitárias, das instalações e dos equipamentos e utensílios utilizados
Proteção	Ações voltadas à garantia da não exposição da comunidade escolar a alimentos não saudáveis, práticas alimentares e situações que representam risco à saúde, assim como a ações ou contextos que promovam ou encorajem, de alguma forma, o consumismo
Ações transversais	Complementam as três vertentes da promoção da alimentação adequada e saudável de diferentes formas. Abarcam: ações de formação continuada dos profissionais; de articulação com famílias e comunidade; de parceria com outros agentes, setores e instâncias; de troca de experiências e informações com outras unidade de Educação Infantil; de monitoramento e de avaliação das iniciativas de incentivo, apoio e proteção da alimentação adequada e saudável; e dos desfechos de saúde, que subsidiam o (re)direcionamento das ações desenvolvidas. Tais ações legitimam, qualificam e fortalecem as ações de incentivo, apoio e proteção da alimentação adequada e saudável

**Quadro 2:** Definições e exemplos dos componentes do modelo teórico de promoção da alimentação adequada e saudável na Educação Infantil de acordo com suas vertentes.

COMPONENTES	DEFINIÇÃO	EXEMPLOS DE PRÁTICAS	AGENTES, SETORES E INSTÂNCIAS ENVOLVIDOS
INCENTIVO			
Inserção da educação alimentar e nutricional como prática transversal ao currículo	Prática de educação alimentar e nutricional incluída como tema transversal e transdisciplinar no currículo escolar, tendo como base as recomendações dos Guias alimentares brasileiros	Adquirir/desenvolver materiais educativos sobre o tema da alimentação adequada e saudável, de acordo com a fase de desenvolvimento da criança	SME e SMS
		Planejar atividades de educação alimentar e nutricional para as crianças com vistas à promoção da amamentação e da alimentação adequada e saudável, à valorização da diversidade e das culturas alimentares e à formação cidadã	Educadores(as), equipe de alimentação escolar, profissionais que realizem ações do PSE e profissionais da UBS do território
		Desenvolver atividades de educação alimentar e nutricional com as crianças de forma lúdica, orgânica e vivencial, por meio da adoção de metodologias ativas, incluindo, por exemplo, a prática culinária, o compartilhamento de receitas dos familiares e com os familiares, e o cultivo de hortas no ambiente escolar	
Incorporação da educação alimentar e nutricional como prática cotidiana	Educação alimentar e nutricional incluída em atividades pedagógicas do cotidiano escolar, para além da sala de aula, como datas festivas, feiras, visitas e reuniões	Garantir coerência entre as rotinas do cotidiano escolar e as abordagens de alimentação adequada e saudável presentes nos documentos orientadores de políticas públicas (como guias alimentares brasileiros e Normas Técnicas do FNDE sobre o tema)	Todos os profissionais da unidade de Educação Infantil
		Aproveitar o momento da oferta de refeições saudáveis baseadas em alimentos *in natura* ou minimamente processados, que contemplem a presença dos diversos grupos de alimentos, para vivências educativas	
Mobilização e formação sobre a promoção da alimentação adequada e saudável junto às famílias	Sensibilização, motivação, educação e orientação das famílias para uma alimentação adequada e saudável por meio de ações de educação alimentar e nutricional	Estimular as mães a continuarem a amamentar antes e depois do período em que as crianças permanecem na unidade de Educação Infantil	Gestor(a) da unidade de Educação Infantil, educadores(as), auxiliares administrativos e profissionais da UBS do território
		Informar às mães/famílias sobre a disponibilidade de sala de amamentação para as mães extraírem o leite na unidade de Educação Infantil	Todos os profissionais da unidade de Educação Infantil
		Orientar e encorajar as mães a manterem a amamentação após a entrada da criança na unidade de Educação Infantil. Oferecer informação às famílias sobre como apoiar a retirada do leite do peito e sobre o seu armazenamento, caso as mães optem por essa prática	Gestor(a) da unidade de Educação Infantil, educadores(as), equipe de alimentação escolar, profissionais da UBS do território e profissionais que realizam ações de PSE
		Criar oportunidades de encontro e compartilhamento entre as famílias, por exemplo, por meio de grupos de discussão e de oficinas práticas formativas e criativas, que utilizem abordagens dialógicas e promovam debates e reflexões sobre alimentação adequada e saudável	
		Reforçar a corresponsabilidade da família e a importância de sua participação no processo do estabelecimento da alimentação adequada e saudável no ambiente escolar	
		Abordar o tema alimentação adequada e saudável nas reuniões com os responsáveis das crianças	
		Realizar atividades de educação alimentar e nutricional junto aos responsáveis das crianças, como oficina de culinária com as famílias	
		Capacitar famílias e cuidadores a tomar as melhores decisões para proteger as crianças contra o marketing de alimentos	
APOIO			
Oferta de leite humano	Abarca ações que visam garantir infraestrutura e rotinas de acolhimento e orientações que permitam às mães/famílias manter a amamentação após o ingresso da criança na unidade de Educação Infantil	Dispor de sala de amamentação, de acordo com as normas, que permita às mães extraírem o seu leite na unidade de Educação Infantil	Gestor(a) da unidade de Educação Infantil, gestão da alimentação escolar no município e SME
		Dispor de infraestrutura e rotinas para recebimento adequados, armazenamento e oferta do leite humano na unidade de Educação Infantil	Gestor(a) da unidade de Educação Infantil e SME
		Apoiar a amamentação, incentivando e orientando as mães sobre a retirada do leite humano, armazenamento em casa e transporte para a unidade de Educação Infantil. Dispor de orientações expostas nas paredes e em panfletos para as famílias sobre a amamentação antes e depois do período em que as crianças permanecem na unidade de Educação Infantil, assim como sobre a retirada do leite humano, armazenamento em casa e transporte para a unidade de Educação Infantil	Gestor(a) da unidade de Educação Infantil educadores(as), auxiliares administrativos e profissionais da UBS do território
		Oferecer leite humano extraído de forma segura à criança, utilizando copinho, copo específico ou de transição, xícara ou colher individual e devidamente higienizado	Educadores(as)
Oferta de alimentação adequada e saudável	Abarca ações que viabilizem infraestrutura e rotinas que garantam a oferta de alimentação adequada e saudável, bem como ações de redução de desperdício e destino adequado de resíduos	Elaborar cardápio baseado em alimentos *in natura* ou minimamente processados, de modo a respeitar as necessidades nutricionais, os hábitos alimentares, a cultura alimentar da localidade e pautar-se na sustentabilidade, sazonalidade e diversificação agrícola da região e na promoção da alimentação adequada e saudável	Equipe de alimentação escolar e Gestão de Alimentação Escolar no município
		Adquirir alimentos adequados e saudáveis, respeitando a cultura alimentar e as necessidades nutricionais das crianças	Gestão de Alimentação Escolar no município e SME
		Adquirir, preferencialmente, alimentos de base agroecológica e produzidos em âmbito local, pela agricultura familiar e pelos produtores familiares rurais, respeitando a cultura local e contribuindo com a sustentabilidade	
		Fiscalizar se pelo menos 30% dos recursos financeiros oriundos do FNDE estão sendo direcionados para a aquisição de alimentos de base agroecológica e produzidos em âmbito local, pela agricultura familiar e pelos produtores familiares rurais	CAE
		Garantir instalações e condições higiênico-sanitárias em conformidade com as legislações vigentes e o uso de utensílios e mobiliário adequados	Gestor(a) da unidade de Educação Infantil, Gestão de Alimentação Escolar no município e SME
		Criar condições para que a alimentação seja oferecida em um ambiente arejado, acolhedor e que comporte o número de crianças atendidas em cada horário durante um tempo suficiente para que as refeições sejam tranquilas	Gestor(a) da unidade de Educação Infantil e SME
		Estabelecer os horários das refeições respeitando o tempo requerido pelas crianças em relação à fome e à saciedade	Gestor(a) da unidade de Educação Infantil
		Adotar a prática da alimentação responsiva: assegurar respeito ao grau de aceitação da criança em relação à alimentação, atender aos sinais de fome e saciedade manifestados pela criança, não forçar a criança a comer	Educadores(as)
		Observar a adequação do tamanho das porções de alimentos servidas	Manipuladores(as) de alimentos
		Disponibilizar o cardápio com as informações nutricionais em locais visíveis na unidade de Educação Infantil e nos sítios eletrônicos oficiais da unidade	Gestor(a) da unidade de Educação Infantil e auxiliares administrativos
		Preparar a alimentação com diferentes consistências para oferecer preparações culinárias compatíveis com os diferentes estágios de desenvolvimento das crianças	Manipuladores(as) de alimentos
		Garantir oferta de água própria para consumo em bebedouros em condições sanitárias adequadas	Gestor(a) da unidade de Educação Infantil
		Propiciar às crianças momentos de alimentação como experiência lúdica e assegurar possibilidade de convivência e socialização durante o compartilhamento da comida	Educadores(as)
		Criar rotinas de monitoramento do desperdício e estratégias para reduzi-lo; garantir o destino adequado de resíduos, com vistas à sustentabilidade	Gestor(a) da unidade de Educação Infantil, equipe de alimentação escolar e Gestão da Alimentação Escolar no município
Atendimento a crianças com necessidades alimentares especiais ou específicas	Engloba ações voltadas para atender as necessidades nutricionais das crianças que apresentam necessidades alimentares especiais (decorrentes de problemas de saúde, por exemplo diabetes, intolerância à lactose, autismo etc.) ou específicas (concepções da família em relação à alimentação, por exemplo)	Implementar protocolo de conduta para os casos de crianças com suspeita e com diagnóstico de necessidades alimentares especiais ou específicas, incluindo concepções da família em relação à alimentação (ex: vegetarianismo)	Gestor(a) da unidade de Educação Infantil, equipe de alimentação escolar e profissionais da UBS do território
		Adaptar o cardápio para que atenda às necessidades nutricionais das crianças que apresentam necessidades alimentares especiais ou específicas durante o período letivo	Equipe de alimentação escolar
		Adquirir gêneros alimentícios a serem usados nas adaptações das preparações para as crianças que possuem necessidades alimentares especiais ou específicas	Gestão da Alimentação Escolar no município
		Solicitar, periodicamente, às famílias de crianças com necessidades alimentares especiais um relatório de saúde realizado por profissionais de saúde	Gestor(a) da unidade de Educação Infantil e auxiliares administrativos
		Elaborar e disponibilizar, periodicamente, às famílias das crianças atendidas relatório de saúde para ser entregue à unidade de Educação Infantil	Profissionais da UBS do território
		Possuir protocolo de oferta de refeições para crianças com suspeita ou diagnóstico de necessidades alimentares especiais ou específicas	Gestor(a) da unidade de Educação Infantil
		Realizar as adaptações necessárias durante a elaboração das refeições das crianças que possuem necessidades alimentares especiais ou específicas	Manipuladores(as) de alimentos
PROTEÇÃO			
Controle higiênico- sanitário da alimentação	Abrange medidas que garantam condições físicas e processos adequados às boas práticas de manipulação de alimentos, além de procedimentos operacionais padronizados, a fim de produção de refeições na unidade de Educação Infantil, ou por empresa terceirizada, e sua oferta/distribuição para as crianças.	Elaborar Manual de Boas Práticas de Manipulação de Alimentos e estabelecer os Procedimentos Operacionais Padronizados para Serviços de Alimentação individualizados para a unidade de Educação Infantil	Equipe de alimentação escolar
		Cumprir com o determinado no Manual de Boas Práticas de Manipulação de Alimentos e nos Procedimentos Operacionais Padronizados para Serviços de Alimentação	Manipuladores(as) de alimentos
		Higienizar a área de preparação de alimentos quantas vezes forem necessárias e imediatamente após o término do trabalho	
		Disponibilizar produtos e utensílios adequados, em conformidade com o determinado em legislação, e em quantidade suficiente para a higienização da área de preparo de alimentação	Gestor(a) da unidade de Educação Infantil e SME
		Fiscalizar irregularidades higiênico-sanitárias nas condições físicas, na manipulação de alimentos, na produção e na oferta de refeições na unidade de Educação Infantil para as crianças	SMS e CAE
Prevenção de acidentes nos ambientes de preparo e oferta de refeições	Refere-se a medidas que visam evitar acidentes com os manipuladores de alimentos e as crianças nos ambientes da cozinha e refeitório	Proporcionar segurança para os(as) manipuladores(as) de alimentos e as crianças, garantindo que as áreas destinadas ao preparo e ao cozimento dos alimentos estejam em conformidade com o determinado em legislação específica, sejam reservadas e de difícil acesso às crianças	Gestor(a) da unidade de Educação Infantil e SME
		Usar EPI disponibilizados pelo empregador durante todo o período de trabalho dentro das áreas destinadas ao preparo e ao cozimento dos alimentos	Manipuladores(as) de alimentos
		Disponibilizar EPI adequados para os(as) manipuladores(as) de alimentos, em conformidade com o determinado em legislação, e em quantidade suficiente	Gestor(a) da unidade de Educação Infantil e SME
		Realizar visitas regulares às unidade de Educação Infantil com vistas à identificação de perigos para os(as) manipuladores(as) de alimentos e as crianças	CAE
Regulação da oferta, do comércio e da publicidade de alimentos ultraprocessados e preparações não recomendadas	Abrange ações que visam evitar, no âmbito da unidade de Educação Infantil, a exposição das crianças à oferta, ao comércio ou à publicidade de alimentos ultraprocessados e preparações não recomendadas, como aquelas com alto teor de sal, açúcar e gordura, cujo consumo apresenta risco para a saúde em curto ou longo prazos. Compreende também a prevenção de estratégias de ação política corporativa, e situações de conflito entre interesses públicos e privados em relação à alimentação	Proibir a oferta de alimentos ultraprocessados e a adição de açúcar, mel e adoçante nas preparações culinárias e bebidas para as crianças até três anos de idade, durante todo o ano letivo, incluindo momentos de eventos, festividades e atividades nos finais de semana, conforme orientações do FNDE	Gestão de Alimentação Escolar no município e SME
		Elaborar normativas que favoreçam a prevenção de situações de conflitos de interesses nas unidade de Educação Infantil	
		Elaborar normativas e orientações acerca de lanches levados de casa para unidade de Educação Infantil	Gestor(a) da unidade de Educação Infantil, equipe de alimentação escolar e gestão de alimentação escolar no município
		Garantir a não exposição das crianças a alimentos ultraprocessados e preparações não recomendadas, ou a ações ou contextos que promovam ou encorajem, de alguma forma, o consumismo	Gestor(a) da unidade de Educação Infantil, educadores(as), gestão de alimentação escolar no município e SME
		Prevenir situações de conflito de interesses, por exemplo: não realizar parcerias com empresas privadas (como as indústrias de alimentos) para, por exemplo, desenvolver atividades educativas com as crianças ou patrocinar atividades (como gincanas); não premiar as crianças com visitas a empresas que produzem alimentos ultraprocessados	
		No caso de a Educação Infantil ser ofertada em unidades escolares mistas que possuam cantina: não dispor de alimentos ultraprocessados e preparações não recomendadas para venda na cantina	Cantineiros(as)
AÇÕES TRANSVERSAIS			
Formação dos profissionais	Abarca sensibilização, capacitação e formação continuada de todos os profissionais da unidade de Educação Infantil para promoção da alimentação adequada e saudável	Realizar atividades de formação permanente com todos os profissionais da unidade de Educação Infantil que objetivem apoiar a reflexão sobre os temas da alimentação adequada e saudável	Gestor(a) da unidade de Educação Infantil, equipe de alimentação escolar, profissionais da UBS do território, profissionais que realizem ações do PSE, Gestão da Alimentação Escolar no município, SME, SMS e universidades
		Realizar atividades de capacitação e sensibilização para os profissionais das unidade de Educação Infantil para promover a amamentação e para atuar na orientação quanto à continuidade dessa prática no ambiente escolar	
		Desenvolver atividades de educação alimentar e nutricional que envolvam todos os profissionais da unidade de Educação Infantil abordando a culinária, a manipulação de alimentos e o papel de educador de cada ator social, considerando a alimentação como constituinte da educação e do cuidado integral	Gestor(a) da unidade de Educação Infantil, equipe de alimentação escolar, profissionais da UBS do território, profissionais que realizem ações do PSE, Gestão da Alimentação Escolar no município, SME e universidades
		Mobilizar a comunidade escolar para que entenda o funcionamento do PNAE e valorize a alimentação escolar	
		Capacitar os(as) manipuladores(as) de alimentos acerca das boas práticas de manipulação de alimentos, com uma frequência mínima anual, de acordo com as necessidades das equipes	Equipe de alimentação escolar, profissionais que realizem ações do PSE, Gestão da Alimentação Escolar no município e SME
		Capacitar os(as) manipuladores(as) de alimentos sempre que forem inseridas preparações novas no cardápio	
Interação com outros agentes, setores ou instâncias	Envolve ações de articulação com as famílias e a comunidade, de parceria com outros setores ou instâncias, e de troca de experiências com outras unidade de Educação Infantil com vistas à promoção da alimentação adequada e saudável	Desenvolver atividades de articulação/integração/interação das famílias e da comunidade com a unidade de Educação Infantil, com objetivo de fortalecimento de vínculos	Gestor(a) da unidade de Educação Infantil e educadores(as)
		Organizar espaços para interação das famílias com profissionais de saúde que atuam na unidade de Educação Infantil, para a realização de atividades educativas sobre alimentação adequada e saudável	Gestor(a) da unidade de Educação Infantil
		Assegurar o direito da família de conhecer, acompanhar e opinar sobre o cardápio oferecido na unidade de Educação Infantil, participando ativamente na alimentação escolar	Gestor(a) da unidade de Educação Infantil e Gestão da Alimentação Escolar no município
		Acompanhar e orientar sobre os horários das refeições servidas nas unidade de Educação Infantil	CAE e COMSEA
		Estabelecer parcerias que fortaleçam e ampliem as ações de promoção da amamentação nas unidade de Educação Infantil	Gestor(a) da unidade de Educação Infantil, educadores(as) e profissionais da UBS do território
		Articular parcerias livres de conflitos de interesses para a promoção da alimentação adequada e saudável na unidade de Educação Infantil	Gestor(a) da unidade de Educação Infantil e educadores(as)
		Oferecer assistência, orientações e sementes para o cultivo de hortas escolares	Secretaria Municipal de Agricultura, Meio Ambiente e agricultores(as) familiares
		Articular parcerias para a implementação de hortas escolares como espaços pedagógicos	Gestor(a) da unidade de Educação Infantil, educadores(as), outras unidade de Educação Infantil e Secretarias Municipais de Agricultura e Meio Ambiente
		Compartilhar informações e vivências de práticas de promoção da alimentação adequada e saudável com outras unidade de Educação Infantil da rede escolar	Gestor(a) da unidade de Educação Infantil, educadores(as) e outras unidade de Educação Infantil
		Realizar ações para aproximar a unidade de Educação Infantil da UBS e do CRAS situados no território, a fim de ampliar e potencializar ações	Gestor(a) da unidade de Educação Infantil, SMS e SMAS
		Manter diálogo permanente com a Gestão/Coordenação da Alimentação Escolar de maneira a ampliar e potencializar o desenvolvimento de ações de educação alimentar e nutricional	Gestor(a) da unidade de Educação Infantil
		Estabelecer canais de comunicação acessíveis entre todos os profissionais, tanto da unidade de Educação Infantil, como da UBS, do CRAS e do Conselho Tutelar do território, para discutir os casos de cada criança matriculada na unidade de Educação Infantil que necessite de alguma atenção especial, principalmente relacionada à sua alimentação	Gestor(a) da unidade de Educação Infantil, profissionais da UBS e do CRAS do território, Conselho Tutelar e COMSEA
		Planejar e realizar ações em colaboração para promover e apoiar a amamentação nas unidade de Educação Infantil	
		Estabelecer articulação intersetorial para realização de ações de promoção da alimentação adequada e saudável	
		Construir uma rede de apoio e proteção social para proteção à infância e apoio à amamentação	Todos os profissionais da unidade de Educação Infantil, famílias, comunidade, COMSEA e entidades da sociedade civil
Monitoramento e avaliação	Abarca: (a) iniciativas que objetivam o fornecimento de informações oportunas sobre um determinado contexto, para subsidiar o planejamento de ações; e (b) iniciativas de avaliação de ações executadas com o propósito de construir um juízo de valor sobre elas e subsidiar consequentes ações cabíveis	Realizar diagnóstico inicial institucional ou do contexto sociocultural na unidade de Educação Infantil, para elaborar o planejamento das ações de promoção da alimentação adequada e saudável	Gestor(a) da unidade de Educação Infantil e Gestão da Alimentação Escolar no município
		Realizar avaliação inicial sobre as práticas alimentares de todas as crianças no ato de sua admissão na unidade de Educação Infantil	Gestor(a) da unidade de Educação Infantil, educadores(as) e auxiliares administrativos
		Realizar avaliação das atividades de educação alimentar e nutricional desenvolvidas com a comunidade escolar na unidade de Educação Infantil	Profissionais que realizaram as atividades e universidades
		Realizar avaliação da efetividade das ações de promoção da alimentação adequada e saudável que são executadas na unidade de Educação Infantil	Gestor(a) da unidade de Educação Infantil, Gestão da Alimentação Escolar no município e universidades
		Realizar avaliação das preparações do cardápio executadas pelos(as) manipuladores(as) de alimentos, sempre que for introduzido no cardápio um alimento novo ou quaisquer outras alterações inovadoras	Equipe de alimentação escolar e CAE
		Aplicar teste de aceitabilidade às crianças, sempre que for introduzido no cardápio um alimento novo ou quaisquer outras alterações inovadoras, no que diz respeito ao preparo, ou para avaliar a aceitação dos cardápios praticados frequentemente	
Monitorar o estado nutricional das crianças em sua admissão na unidade de Educação Infantil e no decorrer do ano letivo.	Profissionais da UBS do território, profissionais que realizem ações do PSE e universidades

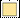
 Práticas de agentes do contexto externo à unidade de Educação Infantil;

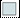
 Práticas de agentes do contexto interno à unidade de Educação Infantil;

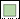
Práticas de responsabilidade compartilhada entre agentes do contexto interno e externo à unidade de Educação Infantil.

CAE: Conselho de Alimentação Escolar; CRAS: Centro de Referência de Assistência Social; COMSEA: Conselho Municipal de Segurança Alimentar e Nutricional; EPI: equipamento de proteção individual; FNDE: Fundo Nacional de Desenvolvimento da Educação; SMAS: Secretaria Municipal de Assistência Social; SME: Secretaria Municipal de Educação; SMS: Secretaria Municipal de Saúde; UBS: unidade básica de saúde; PNAE: Programa Nacional de Alimentação Escolar; PSE: Programa Saúde na Escola.

Nota: todos os profissionais da unidade de Educação Infantil - gestor(a), educadores(as), manipuladores(as) de alimentos, auxiliares administrativos, auxiliares de serviços gerais/apoio, orientadores(as) pedagógicos(as), orientadores(as) educacionais, porteiros(as); comunidade escolar - crianças e seus familiares, equipe gestora da unidade Educação Infantil, educadores(as), manipuladores(as) de alimentos, auxiliares administrativos, auxiliares de serviços gerais/apoio, orientadores(as) pedagógicos(as), orientadores(as) educacionais, porteiros(as), cantineiros(as).

O esquema gráfico (Figura 1) é representado pela forma circular e é dividido em contexto interno e externo à unidade de Educação Infantil; seu núcleo na cor azul escuro representa o objeto central do modelo - promoção da alimentação adequada e saudável na Educação Infantil - circundado pela área que representa a comunidade escolar na cor azul médio, que é constituída pelos agentes do contexto interno à unidade de Educação Infantil. O contexto interno à unidade de Educação Infantil é dividido em três partes iguais, representadas em três tons diferentes da cor azul, referentes às três vertentes de promoção da alimentação adequada e saudável na Educação Infantil que se interconectam (incentivo, apoio e proteção), em que estão dispostos os componentes do modelo teórico. As vertentes de promoção da alimentação adequada e saudável na Educação Infantil encontram-se circundadas pela área referente às ações transversais, representada pela cor verde, que complementa as vertentes, e em que estão dispostos os demais componentes do modelo.

O contexto externo à unidade de Educação Infantil se encontra na área mais externa do círculo. Ele circunscreve-se ao âmbito do município, ou seja, foge ao escopo deste estudo a abordagem das esferas de Governo Estadual e Federal. Ele encontra-se ordenado em dois círculos concêntricos, representados pela cor amarela em degradê e se divide em: contexto externo proximal (amarelo escuro), que conta com agentes, setores e instâncias presentes no território próximo à unidade de Educação Infantil (exemplo: unidade básica de saúde do território), e contexto externo distal (amarelo claro), composto por agentes, setores e instâncias do nível municipal, porém mais distantes da unidade de Educação Infantil (exemplos: Secretaria Municipal de Saúde e universidades públicas e privadas). Esse último nível aglutina agentes, setores e instâncias de nível regional e central.

As cores do esquema gráfico foram assim escolhidas para facilitar a compreensão dos elementos estruturantes do modelo. As áreas onde se encontram as ações de promoção da alimentação adequada e saudável na Educação Infantil, divididas por suas respectivas vertentes, estão representadas por tons diferentes da cor azul, que caracteriza o contexto interno à unidade de Educação Infantil, pois é nesse contexto que são concretizadas. A área em que se encontra a comunidade escolar é representada por um tom de azul, que é uma cor primária, por se tratarem dos agentes do contexto interno à unidade de Educação Infantil. A área em que se encontram os agentes, setores e instâncias do contexto externo à unidade de Educação Infantil é representada pela cor amarela em degradê, outra cor primária. A área que apresenta as ações transversais é representada pela cor verde, uma cor secundária oriunda da junção das cores azul e amarela, com o sentido de que essas são ações que pressupõem a corresponsabilidade dos agentes dos contextos interno e externo à unidade de Educação Infantil, para serem executadas na unidade de Educação Infantil.

Todos os agentes, setores e instâncias envolvidos direta ou indiretamente na promoção da alimentação adequada e saudável na Educação Infantil, tanto do contexto interno quanto do contexto externo à unidade de Educação Infantil, encontram-se com recurso gráfico de fonte diferenciada das ações e dos demais elementos estruturantes do esquema gráfico, com o intuito de destacá-los. Os termos “contexto interno à unidade de Educação Infantil” e “contexto externo à unidade de Educação Infantil” também são apresentados com fonte e cor diferenciadas. Esses dois termos encontram-se sobre as linhas tracejadas que limitam as respectivas áreas, a fim de expressar a interligação entre os contextos, incluindo os seus agentes e as ações por eles executadas.

No Quadro 1, são detalhadas as descrições de cada elemento estruturante do modelo teórico: os contextos interno e externo à unidade de Educação Infantil, seus respectivos agentes, e as vertentes de promoção da alimentação adequada e saudável. No Quadro 2, a primeira coluna apresenta os componentes do modelo teórico e a segunda coluna, suas respectivas definições. Na terceira coluna, são apresentadas algumas formas de concretização das ações de promoção da alimentação adequada e saudável por agentes, setores e instâncias envolvidos na Educação Infantil, isto é, um conjunto não exaustivo de exemplos de práticas de diferentes naturezas, com objetivo de fornecer um amplo escopo de possibilidades para favorecer a promoção da alimentação adequada e saudável nesse âmbito.

Na última coluna do Quadro 2, são apresentados exemplos de agentes, setores e instâncias envolvidos na promoção da alimentação adequada e saudável na Educação Infantil, na seguinte ordem: primeiro os pertencentes ao contexto interno, depois, os pertencentes ao contexto externo à unidade de Educação Infantil. Os exemplos de práticas e de agentes, setores e instâncias envolvidos referentes ao contexto interno à unidade de Educação Infantil encontram-se nas células de cor azul, e os pertencentes ao contexto externo à unidade de Educação Infantil encontram-se nas células de cor amarela. Já os exemplos de práticas que são de responsabilidade compartilhada entre os agentes, setores e instâncias de ambos os contextos encontram-se nas células de cor verde, pois essas práticas não dependem exclusivamente da gestão escolar, e podem envolver questões que fogem a sua capacidade de gerência, como infraestrutura e financiamento.

Uma vez apresentado o modelo teórico, cabem alguns comentários. Esse modelo tem a intenção de expressar a realidade brasileira, tendo em vista que este país possui um arcabouço de políticas públicas nacionais que se expressam em nível local. Nesse sentido, considerando que os sistemas de ensino público e privado são distintos em diversos aspectos, optou-se por elaborar o modelo voltado ao âmbito do ensino público, com objetivo de focar em questões ligadas à promoção da alimentação adequada e saudável na Educação Infantil nesse contexto. Essa escolha se deu tendo em vista as políticas públicas que dialogam com a promoção da alimentação adequada e saudável no âmbito da rede pública de ensino, por exemplo, o PNAE e o Programa Saúde na Escola.

Espera-se que, no processo de promoção da alimentação adequada e saudável na Educação Infantil, as prefeituras se reconheçam nesse modelo teórico e possam identificar estratégias para o fortalecimento de suas ações. O modelo não tem a intenção de ser universal, pois a elaboração de um modelo universal requereria abdicar de especificidades do contexto brasileiro, por exemplo, os agentes envolvidos e as políticas públicas. Entretanto, esse modelo pode ser adaptado para realidades de outros países e para o sistema de ensino privado.

Entende-se que a promoção da amamentação é um elemento da promoção da alimentação adequada e saudável. Entretanto, no processo de elaboração do modelo teórico, optou-se por destacá-la como uma prática “em si”, tendo em vista as particularidades das rotinas nela envolvidas. As ações indicadas no modelo proposto precisam ser planejadas e executadas considerando as singularidadesdos grupos etários que constituem a Educação Infantil (creche: bebês - 0 a 1 ano e 6 meses - e crianças bem pequenas - 1 ano e 7 meses a 3 anos e 11 meses -, e pré-escola: crianças pequenas - 4 anos a 5 anos e 11 meses).

Os termos “agente(s)”, “contexto interno à unidade de Educação Infantil” e “contexto externo à unidade de Educação Infantil” consideram os elementos do referencial teórico-conceitual da TEF. A expressão “comunidade escolar” compreende os agentes envolvidos em todos os processos educativos e rotinas da criança na unidade de Educação Infantil, incluindo ela mesma. Portanto, ela é constituída pelos(as) alunos(as), seus familiares, todos os profissionais da unidade de Educação Infantil e também os(as) cantineiros(as), nos casos em que a Educação Infantil é oferecida em unidades escolares mistas, com mais de um segmento de ensino, que dispõem de cantinas. A expressão “todos os profissionais da unidade de Educação Infantil” compreende todos os profissionais que trabalham na unidade de Educação Infantil, incluindo: equipe gestora da unidade de Educação Infantil, educadores(as), manipuladores(as) de alimentos, auxiliares administrativos, auxiliares de serviços gerais/apoio, orientadores(as) pedagógicos(as) e educacionais, porteiros(as) e outros trabalhadores que, porventura, atuem na unidade de Educação Infantil. Todos os profissionais da unidade de Educação Infantil são agentes protagonistas no processo de promoção da alimentação adequada e saudável, assim como as crianças também o são.

O termo “educadores(as)” compreende os(as) profissionais da unidade de Educação Infantil que atuam diretamente no processo de ensino-aprendizagem junto aos alunos, por exemplo, os(as) professores(as), cuidadores(as) e estimuladores(as). O termo “manipuladores(as) de alimentos” compreende os(as) profissionais da unidade de Educação Infantil que trabalham nas áreas destinadas ao preparo e à distribuição das refeições oferecidas pela unidade de Educação Infantil. Esse termo tem o mesmo sentido de outros que costumam ser utilizados tanto na literatura quanto no cotidiano escolar, por exemplo, “merendeiros(as)”, “auxiliares de cozinha” e “cozinheiros(as) escolares”.

### Validação de face e de conteúdo do modelo teórico

As etapas de sistematização do levantamento bibliográfico e de validação de face e de conteúdo foram fundamentais para o desenvolvimento do modelo teórico de promoção da alimentação adequada e saudável na Educação Infantil. Em relação à validade de face, o material empírico produzido nos painéis de especialistas foi sistematizado, examinado à luz do referencial adotado e incorporado no modelo pelas autoras. Quanto à validação de conteúdo e à clareza do modelo teórico, a maioria dos aspectos avaliados foi considerada altamente relevante e clara. O Quadro 3 apresenta os resultados da validação de conteúdo. Os aspectos que obtiveram valor de CVR mais baixos foram referentes ao esquema gráfico: o componente “Atuação em casos de necessidades alimentares especiais”, pertencente à vertente apoio, e os “Agentes do contexto externo à unidade de Educação Infantil”, ambos com valor de 0,8. Nenhum aspecto avaliado foi descartado, uma vez que todos apresentaram valor de CVR superior ao ponto de corte adotado (0,62). O modelo foi aprimorado incorporando-se as sugestões das especialistas, sempre que consideradas pertinentes e oportunas pelas autoras.

**Quadro 3:** Validação de conteúdo dos aspectos do modelo teórico de promoção da alimentação adequada e saudável na Educação Infantil.

		CVR *	QUANTIDADE DE SUGESTÕES DE ESCRITA	SUGESTÕES	VERSÃO APÓS A SUGESTÃO DE REESCRITA
ESQUEMA GRÁFICO DO MODELO TEÓRICO					
Incentivo	Educação alimentar e nutricional como prática cotidiana	1,0	0	-	-
	Inclusão da educação alimentar e nutricional como tema transversal do currículo	1,0	2	Educação alimentar e nutricional como prática transversal ao currículo	“Educação alimentar e nutricional como prática transversal ao currículo”
	Mobilização e formação das famílias para a promoção da alimentação adequada e saudável	1,0	3	Sensibilização e diálogo para a promoção da alimentação adequada e saudável junto às famílias O termo formação remete muitas vezes a um papel mais passivo. Sugestão: capacitação	“Mobilização e formação sobre a promoção da alimentação adequada e saudável junto às famílias”
Apoio	Favorecimento da manutenção do aleitamento materno	1,0	5	A ideia de “favorecimento” já está implícita em “Apoio”; sugestão: manter somente “Manutenção do aleitamento materno” Substituir a sigla por amamentação Promoção do aleitamento materno	“Manutenção da amamentação”
	Oferta de alimentação adequada e saudável	1,0	0	-	-
	Atuação em casos de necessidades alimentares especiais	0,8	5	Atendimento a (ou das) necessidades alimentares especiais O termo “necessidades especiais” remete para necessidades decorrentes de problemas de saúde. Incluir também necessidades específicas que remetam, por exemplo, para preferências ou ideologia da família em relação à alimentação	“Atendimento a crianças com necessidades alimentares especiais ou específicas”
Proteção	Garantia de controle higiênico-sanitário da alimentação	1,0	1	Suprimir a palavra “Garantia”	“Controle higiênico-sanitário da alimentação”
	Prevenção de acidentes nos ambientes de preparo e oferta de refeições	1,0	2	Garantia da infraestrutura e condições adequadas de trabalho	Sem alteração
	Controle de oferta, comércio e promoção de alimentos ultraprocessados e preparações não recomendadas	1,0	2	Regulamentação da oferta, do comércio e da publicidade de alimentos ultraprocessados	“Regulação da oferta, do comércio e da publicidade de alimentos ultraprocessados e preparações não recomendadas”
Ações transver sais	Formação dos profissionais	1,0	0	-	-
	Interação com outros agentes, setores e instâncias	1,0	0	-	-
	Avaliação	1,0	3	Monitoramento e avaliação Acompanhamento e avaliação das ações e estratégias de promoção da alimentação adequada e saudável Avaliação e acompanhamento das ações e estratégias de promoção da alimentação adequada e saudável	“Monitoramento e avaliação”
Agentes do contexto interno à unidade de Educação Infantil (comunidade escolar)		1,0	3	Incluir toda a equipe gestora como agente nesse contexto	“Equipe gestora da unidade de Educação Infantil”
				Adotar o termo atores sociais para as ações de educação alimentar e nutricional no PNAE, corrobora a ideia de corresponsabilidade	Sem alteração
				As famílias de escolares aparecem tanto em agentes externos como em agentes internos; manteria em agentes internos	Sem alteração
Agentes do contexto externo à unidade de Educação Infantil		0,8		Incluir o CECANE	Sem alteração
Representações gráficas	Vertentes de promoção da alimentação adequada e saudável (incentivo, apoio, proteção e ações transversais) em relação à escrita, forma e disposição gráfica	1,0	1	No centro do modelo, sugiro manter somente “Educação Infantil” ou “escolares” ou “crianças”	Sem alteração
	Componentes do modelo (em relação à escrita, forma e disposição gráfica)	1,0	2	-	-
	Contextos interno e externo à unidades de Educação Infantil (em relação à escrita, forma e disposição gráfica)	1,0	0	-	-
	Agentes do contexto interno à unidade de Educação Infantil	1,0	0	-	-
	Agentes do contexto externo à unidade de Educação Infantil	1,0	2	Atores ao invés de agentes Iniciar a expressão “outras unidades de Educação Infantil” com caixa alta, como nos outros casos	Sem alteração “Outras unidades de Educação Infantil”
	Cores usadas	1,0	0	-	-
QUADRO DE DESCRIÇÃO DOS ELEMENTOS ESTRUTURANTES DO MODELO TEÓRICO					
Contexto interno à unidade de Educação Infantil		1,0	0	-	-
Contexto externo à unidade de Educação Infantil		1,0	1	Incluir o CECANE	“...universidades públicas e privadas - incluindo os CECANE”
Agentes do contexto interno à unidade de Educação Infantil		1,0	0	-	-
Agentes do contexto externo à unidade de Educação Infantil		1,0	1	Incluir ONGs. Existem aquelas que defendem e lutam pela oferta de alimentação adequada e saudável nas escolas	“Contexto externo proximal: famílias das crianças atendidas na unidade de Educação Infantil, (…) e entidades da sociedade civil”
Vertente de incentivo		1,0	0	-	-
Vertente de apoio		1,0	0	-	-
Vertente de proteção		1,0	0	-	-
Ações transversais		1,0	0	-	-
QUADRO DE DEFINIÇÕES E EXEMPLOS DOS COMPONENTES DO MODELO TEÓRICO					
Definições					
Incentivo	Educação alimentar e nutricional como prática cotidiana	1,0	0	-	-
	Inclusão da educação alimentar e nutriconal como tema transversal do currículo	1,0	2	Resumir textos Substituir “compartilhamento de receitas familiares” por “compartilhamento de receitas na família”	Sem alteração “...compartilhamento de receitas dos familiares e com os familiares...”
	Mobilização e formação sobre a promoção da alimentação adequada e saudável junto às famílias	1,0	1	Incluir entre os responsáveis “profissionais que realizam ações de PSE”. Os responsáveis podem aproveitar a presença desses profissionais para sanar estas e outras dúvidas referentes à alimentação infantil	“Gestor(a) da unidade de Educação Infantil, educadores(as), equipe de alimentação escolar, profissionais da UBS do território e profissionais que realizem ações de PSE”
Apoio	Favorecimento da manutenção do aleitamento materno	1,0	0	-	-
	Oferta de alimentação adequada e saudável	1,0	0	-	-
	Atuação em casos de necessidades alimentares especiais	1,0	0	-	-
Proteção	Garantia de controle higiênico- sanitário da alimentação	1,0	1	Inclui alimentação trazida para escola por estudantes? Se forem somente as refeições produzidas na escola, sugiro substituir por “controle higiênico-sanitário da produção de refeições”	“Abrange medidas que garantam condições físicas e processos adequados às boas práticas de manipulação de alimentos, além de procedimentos operacionais padronizados, a fim de produção de refeições na unidade de Educação Infantil, ou por empresa terceirizada, e sua oferta/distribuição para as crianças”
	Prevenção de acidentes nos ambientes de preparo e oferta de refeições	1,0	0	-	-
	Controle de oferta, comércio e promoção de alimentos ultraprocessados e preparações não recomendadas	1,0	0	-	-
Ações transver sais	Formação dos profissionais	1,0	0	-	-
	Interação com outros agentes, setores e instâncias	1,0	0	-	-
	Avaliação	1,0	0	-	-
Exemplos de práticas					
Incentivo	Educação alimentar e nutricional como prática cotidiana	1,0	0	-	-
	Inclusão da educação alimentar e nutricional como tema transversal do currículo	1,0	0	-	-
	Mobilização e formação das famílias para a promoção da alimentação adequada e saudável	1,0	0	-	-
Apoio	Favorecimento da manutenção do aleitamento materno	1,0	2	Substituir “que permita às mães extraírem o leite…” por “que permitam às mães extraírem o seu leite humano” Substituir “Oferecer leite materno retirado de forma segura à criança” por “Oferecer leite humano extraído ou ordenhado de forma segura à criança (binômio mãe filho)” A maioria dos exemplos remete efetivamente para uma perspectiva de ambientes de suporte (condições para a manutenção do aleitamento materno). Mas há alguns exemplos que remetem mais para ações de educação para a saúde, que incentivem as mães a amamentar. Nesse caso, parece-me haver sobreposição com a vertente do incentivo, no componente de mobilização e formação	“Dispor de sala de amamentação, de acordo com as normas, que permita às mães extraírem o seu leite na unidade de Educação Infantil” “Oferecer leite humano extraído ou ordenhado de forma segura à criança, utilizando copinho, copo específico ou de transição, xícara ou colher individual e devidamente higienizado” Exemplos movidos do componente “Manutenção da amamentação”, com suas respectivas sugestões de exemplos de agentes, setores e instâncias envolvidos: “Estimular as mães a continuarem a amamentar antes e depois do período em que as crianças permanecem na unidade de Educação Infantil” e “Informar às mães/famílias sobre a disponibilidade de sala de amamentação para as mães extraírem o leite na unidade de Educação Infantil”
	Oferta de alimentação adequada e saudável	1,0	0	-	-
	Atuação em casos de necessidades alimentares especiais	1,0	0	-	-
Proteção	Garantia de controle higiênico-sanitário da alimentação	1,0	0	-	-
	Prevenção de acidentes nos ambientes de preparo e oferta de refeições	1,0	1	Substituir o exemplo “Usar calças compridas e sapatos fechados durante todo o período de trabalho dentro das áreas destinadas ao preparo e ao cozimento dos alimentos” por “Utilizar EPI disponibilizados pelo empregador” ou por “Participar de atividades de formação sobre uso seguro de equipamentos”	“Usar EPI disponibilizados pelo empregador durante todo o período de trabalho, dentro das áreas destinadas ao preparo e ao cozimento dos alimentos”
	Controle de oferta, comércio e promoção de alimentos ultraprocessados e preparações não recomendadas	1,0	0	-	-
Ações transver sais	Formação dos profissionais	1,0	1	Substituir “Realizar atividades de capacitação para os profissionais das unidade de Educação Infantil para promover o aleitamento materno” por “Realizar atividades de capacitação e sensibilização para os profissionais...”	“Realizar atividades de capacitação e sensibilização para os profissionais das unidade de Educação Infantil para promover a amamentação e para atuar na orientação quanto à continuidade desta prática no ambiente escolar”
	Interação com outros agentes, setores e instâncias	1,0	0	-	-
	Avaliação	1,0	0	-	-
Sugestões de agentes envolvidos nas práticas					
Incentivo	Educação alimentar e nutricional como prática cotidiana	1,0	0	-	-
	Inclusão da Educação alimentar e nutricional como tema transversal do currículo	1,0	0	-	-
	Mobilização e formação das famílias para a promoção da alimentação adequada e saudável	1,0	0	-	-
Apoio	Favorecimento da manutenção do aleitamento materno	1,0	0	-	-
	Oferta de alimentação adequada e saudável	1,0	0	-	-
	Atuação em casos de necessidades alimentares especiais	1,0	0	-	-
Proteção	Garantia de controle higiênico- sanitário da alimentação	1,0	0	-	-
	Prevenção de acidentes nos ambientes de preparo e oferta de refeições	1,0	0	-	-
	Controle de oferta, comércio e promoção de alimentos ultraprocessados e preparações não recomendadas	1,0	0	-	-
Ações transver sais	Formação dos profissionais	1,0	0	-	-
	Interação com outros agentes, setores e instâncias	1,0	0	-	-
	Avaliação	1,0	0	-	-

CECANE: Centro Colaborador em Alimentação Escolar; CVR: *content validity ratio*; EPI: equipamento de proteção individual; ONGs: Organizações Não-governamentais; PNAE: Programa Nacional de Alimentação Escolar; PSE: Programa Saúde na Escola.

* CVR = (número de especialistas que classificaram cada aspecto como “essencial” (4 ou 3) - número total de respondentes / 2) / número total de respondentes / 2.

## Discussão

Considerando a natureza teórico-conceitual deste estudo, optou-se, nesta seção, pela reflexão sobre o diálogo dos referenciais teóricos adotados no estudo com o modelo de promoção da alimentação adequada e saudável na Educação Infantil desenvolvido.

### Vertentes de promoção da alimentação adequada e saudável

A análise de políticas e programas voltados à promoção da saúde tem mostrado que as iniciativas que alcançam resultados mais favoráveis têm em comum a articulação de ações intersetoriais sinérgicas e complementares, referentes à combinação de três vertentes de atuação: incentivo, apoio e proteção. Com base nisso, o conceito de promoção da alimentação adequada e saudável se amplia e extrapola a perspectiva de saúde dos indivíduos e coletividades (prevenir doenças, garantir crescimento físico etc.), passando a incorporar noções de garantia do Direito Humano à Alimentação Adequada, resgate e valorização da cultura e da subjetividade na relação com a comida, problematização do consumismo, fortalecimento da cidadania, sustentabilidade ambiental, justiça social, entre outros ^8^.

Nesse contexto, a promoção da alimentação adequada e saudável objetiva a formação de hábitos alimentares saudáveis, a promoção da segurança alimentar e nutricional, o desenvolvimento de habilidades pessoais e a melhoria da qualidade de vida da população por meio de ações, para além do setor Saúde, voltadas ao coletivo, aos indivíduos e aos ambientes alimentares, e fundamentadas nas vertentes de incentivo, apoio e proteção de escolhas, práticas e hábitos alimentares saudáveis, que sejam social, cultural e ambientalmente sustentáveis ^1^.

Para a promoção da alimentação adequada e saudável, a compreensão sobre a interação entre o contexto e as práticas individuais é estruturante dos processos de reflexão e de mudanças. A unidade de Educação Infantil oferece uma gama de possibilidades de interação com as famílias das crianças nela inseridas, suas práticas e seus contextos. Para que as unidades de Educação Infantil sejam espaços promotores da alimentação adequada e saudável, é fundamental abranger a complexidade do conceito de alimentação e suas diferentes dimensões, e desenvolver ações alinhadas às três vertentes de ação ^8^ ^,^ ^9^.

O referencial teórico-conceitual das vertentes da promoção da alimentação adequada e saudável foi a base para o modelo desenvolvido, isto é, as vertentes de promoção da alimentação adequada e saudável foram aplicadas ao contexto da Educação Infantil. Com isso, o modelo teórico se configura como a expressão desse referencial no âmbito da Educação Infantil.

A vertente de incentivo abarca fundamentalmente ações de difusão de informação e de motivação da comunidade escolar para a adoção de hábitos alimentares saudáveis, seu empoderamento e geração de autonomia para lidar, em seu cotidiano, com as questões que envolvem os determinantes de sua saúde relacionados à alimentação. As ações de incentivo podem ser inseridas na Educação Infantil por meio da incorporação dos temas educação alimentar e nutricional e alimentação adequada e saudável, incluindo a cultura da amamentação, de forma transversal no currículo escolar e no projeto político pedagógico, e de ações educativas cotidianas e continuadas com toda a comunidade escolar, tendo como referência o eixo transversal dos parâmetros curriculares sobre saúde. As ações de incentivo devem ter como pressupostos básicos a alimentação como direito humano; a segurança alimentar e nutricional e o estímulo à geração de autonomia e participação ativa dos sujeitos e da comunidade escolar no controle de suas condições de alimentação e saúde. São fundamentais, nesse processo, que sejam resgatadas as dimensões histórica e cultural da alimentação, e que haja investimentos em atividades e instrumentos que subsidiem o educador na abordagem deste tema ^2^ ^,^ ^7^ ^,^ ^8^ ^,^ ^9^ ^,^ ^15^ ^,^ ^16^ ^,^ ^17^.

A vertente de apoio engloba todas as medidas que visam a tornar factíveis e/ou a facilitar a adesão a práticas alimentares saudáveis pela comunidade escolar informada e motivada. Na rede pública de ensino, essa vertente compreende ações que garantam a oferta de água potável, de leite humano e de alimentos e preparações adequadas e saudáveis às crianças, por meio do PNAE, respeitando-se a sua fase de desenvolvimento, a cultura alimentar e as necessidades nutricionais, incluindo as especiais e específicas ^3^ ^,^ ^6^ ^,^ ^7^. Tais garantias devem levar em consideração as condições higiênico-sanitárias recomendadas, o uso de utensílios e instalações adequados, e que a alimentação escolar é direito do aluno e favorece o seu processo de crescimento, desenvolvimento biopsicossocial, aprendizagem, rendimento escolar e formação de práticas alimentares saudáveis. O planejamento dos cardápios deve seguir as diretrizes do PNAE ^3^ ^,^ ^6^ e os guias alimentares brasileiros ^16^ ^,^ ^17^, respeitando-se as recomendações nutricionais para as diferentes faixas etárias atendidas, bem como os hábitos alimentares regionais e a safra de hortaliças e frutas ^8^.

A vertente de proteção abrange iniciativas voltadas a evitar práticas alimentares inadequadas no ambiente da unidade de Educação Infantil, caracterizando-se por medidas que garantam a não exposição da comunidade escolar a: alimentos não saudáveis, práticas alimentares e fatores e situações que representam risco à saúde, como a oferta, comércio e promoção de alimentos ultraprocessados e de preparações com alto teor de sódio, açúcares e gorduras nas dependências e no entorno da unidade de Educação Infantil; barreiras para a amamentação; situações de conflito entre interesses públicos e privados (no sentido de interesses comerciais), assim como a ações ou contextos que promovam ou encorajem, de alguma forma, o consumismo. Isso pode envolver medidas regulatórias, como legislações que restrinjam a venda e a propaganda de alimentos ultraprocessados nas escolas, mas também podem envolver práticas que sejam pactuadas em diálogo com a comunidade escolar, por exemplo, o estabelecimento da regra de não inclusão desses alimentos nos lanches trazidos de casa ou nas comemorações realizadas pelos responsáveis ou professores ^7^ ^,^ ^8^ ^,^ ^9^.

A prática cotidiana de promoção da alimentação adequada e saudável no ambiente escolar da Educação Infantil pressupõe a integração dessas vertentes, complementadas por ações transversais de formação continuada de todos os profissionais da unidade de Educação Infantil; interação entre agentes, setores e instâncias; monitoramento e avaliação das iniciativas de incentivo, apoio e proteção à alimentação adequada e saudável e dos processos e desfechos de saúde, que objetivam subsidiar o (re)direcionamento de políticas e programas ^8^.

Portanto, para a unidade de Educação Infantil se consolidar como espaço promotor da alimentação adequada e saudável, ela deve ser capaz de se organizar para a realização de estratégias que se complementam e que devem ser implementadas de maneira articulada ao cotidiano escolar e sensível à realidade local. Vale destacar que a realização das ações de promoção da alimentação adequada e saudável na Educação Infantil envolve a corresponsabilidade dos agentes da comunidade escolar, internos à unidade de Educação Infantil, e de outros agentes, setores e instâncias externos à unidade de Educação Infantil, como os profissionais da saúde das unidades da atenção primária do território, entre outros. O ambiente alimentar saudável na unidade de Educação Infantil implica a integração de saberes, olhares e ações que favoreçam a apreensão, a reflexão e a vivência da alimentação adequada e saudável ^9^.

### Teoria da Estruturação Forte

Sob o aspecto organizacional, a Educação Básica compõe um sistema social que abarca a Educação Infantil. O ambiente da Educação Infantil é institucionalizado e integra parte da rede física de serviços. Os comportamentos incorporados e reproduzidos por agentes humanos nesse ambiente, no caso a comunidade escolar, é fortemente influenciado por aspectos como regulamentos, normas, códigos de prática profissional e tradições profundamente arraigadas ^18^.

A TEF trata estrutura e agência de forma indissociável. Estas se deslocam do campo abstrato para situações específicas concretas, respondendo questões sobre por que, como e o que da vida cotidiana acontece por meio das disposições e práticas dos agentes ^19^.

As estruturas externas ao agente em foco, primeiro elemento do ciclo quadripartido da estruturação, dizem respeito às condições de ação, como o contexto físico e social ou econômico no qual a ação ocorre ^12^. Elas são concebidas no modelo teórico como contextos externo e interno à unidade de Educação Infantil. O primeiro abarca: as condições do território; o tecido social; o nível de articulação que os diferentes agentes, instituições, organizações, setores e instâncias que interagem com a unidade de Educação Infantil estabelecem e mantêm entre si, circunscritas, no caso do modelo desenvolvido, ao âmbito do município. O segundo engloba o espaço físico e o contexto institucional da unidade de Educação Infantil propriamente dita.

Os agentes em foco são concebidos no modelo como agentes do contexto interno à unidade de Educação Infantil, isto é, as pessoas que compõem a comunidade escolar. Inerentes aos agentes em foco, no ciclo da estruturação, encontram-se as estruturas internas do agente, que representam o segundo elemento do ciclo quadripartido, e a agência ativa ou práticas dos agentes, o terceiro elemento desse ciclo. Stones ^12^ discerne analiticamente dois tipos de estruturas internas pertinentes ao agente: conhecimento conjunturalmente específico e disposições gerais.

O conhecimento conjunturalmente específico sobre regras, convenções, obrigações e normas sociais, adquirido pelos agentes em foco ao longo do tempo, podem envolver entendimentos parciais e experiências passadas. Ele diz respeito à posição ocupada, aos esquemas interpretativos, às capacidades de poder e às expectativas normativas desses agentes dentro do contexto. As disposições gerais dos agentes são constituídas por suas habilidades, ambições, atitudes, valores, experiências passadas e formas de ver o mundo. Tais estruturas orientam a conduta humana em situações específicas no presente, baseando-se na compreensão que os agentes em foco têm das estruturas externas ^12^ ^,^ ^19^.

A agência ativa diz respeito à maneira pela qual um agente em foco age, seja rotineiramente, pré-reflexivamente, estrategicamente ou criticamente, com base nos seus conhecimentos conjunturalmente específicos e nas suas disposições gerais ^12^. A agência ativa é concebida no modelo desenvolvido por meio dos seus componentes, isto é, das ações de incentivo, apoio e proteção da promoção da alimentação adequada e saudável desenvolvidas pelos agentes, complementadas por ações transversais.

Apesar de todos os agentes apontados no modelo teórico terem uma missão convergente com a causa em questão - promoção da alimentação adequada e saudável na Educação Infantil -, as ações de agentes externos à unidade de Educação Infantil (como a equipe de alimentação escolar e as Secretarias Municipais de Educação e Saúde) podem influenciar as ações dos agentes em foco, de forma independente, positiva ou negativamente. De igual modo, os agentes em foco podem escolher não desempenhar seu papel na promoção da alimentação adequada e saudável no âmbito da unidade de Educação Infantil. Vale destacar, ainda, que, no presente estudo, não foram incluídos entre os agentes aqueles antagonistas (por exemplo, indústrias de alimentos que desenvolvem ações de marketing de seus produtos nas escolas), pois objetivou-se elaborar um modelo teórico propositivo de um contexto convergente com as recomendações presentes nos marcos de políticas públicas.

O último elemento do ciclo quadripartido da estruturação são os resultados da agência ativa. Os efeitos das práticas dos agentes em foco afetam ambas as estruturas (interna e externa ao agente) e o modo como elas se configuram. As estruturas podem ser mantidas, alteradas, elaboradas ou reproduzidas; facilitar ou frustrar os propósitos dos agentes em foco, e as consequências podem ser intencionais ou não ^12^ ^,^ ^19^. Os resultados da agência ativa são concebidos no modelo como aspectos da promoção da alimentação adequada e saudável a serem monitorados e avaliados ao longo do tempo, por exemplo: efetividade das ações de promoção da alimentação adequada e saudável realizadas; aceitabilidade da alimentação escolar; práticas alimentares; e estado nutricional das crianças.

A TEF contribuiu na elaboração do modelo teórico, principalmente no sentido de apresentar uma nova perspectiva para as concepções dos elementos constitutivos do modelo, por meio dos seus conceitos, especialmente de agente em foco e de estruturas internas e externas ao agente em foco. Embora o âmbito da Educação Infantil não seja um contexto específico de uma dada realidade (cenário em que comumente essa teoria é adotada ^20^ ^,^ ^21^), ele apresenta elementos próprios para os quais essa teoria pode ser aplicada.

## Conclusão

O modelo teórico de promoção da alimentação adequada e saudável na Educação Infantil consiste em um esquema visual que objetiva organizar as relações existentes entre os conceitos, diretrizes e recomendações sobre esse tema, no qual estão expressos os componentes necessários para o desenvolvimento de ações de promoção da alimentação adequada e saudável no âmbito da Educação Infantil, segundo as suas vertentes de ação, considerando os contextos interno e externo à unidade de Educação Infantil. Ainda que concebido para o contexto da rede pública de ensino brasileira, ele é adaptável a outras realidades.

O modelo teórico aqui proposto contribui para a compreensão sobre promoção da alimentação adequada e saudável na Educação Infantil ao explicitar o escopo das ações da unidade de Educação Infantil concernentes à promoção da alimentação adequada e saudável e a sua articulação com as famílias, a comunidade e outros agentes, setores e instâncias, como a Saúde, criando processos de integração, participação e educação. O modelo também destaca a inclusão da educação alimentar e nutricional como prática cotidiana e transversal ao currículo escolar da Educação Infantil, e valoriza que a estrutura e o funcionamento das unidades de Educação Infantil devem garantir que esse seja um espaço de educação coletiva, com objetivo de promover o desenvolvimento integral das crianças e assegurar seus direitos.

Outra contribuição desse modelo pode ser sua aplicação em estudos empíricos que busquem compreender os contextos por meio das percepções dos agentes à luz da TEF. Estudos que objetivem compreender as relações existentes nos contextos interno e externo à unidade de Educação Infantil e entre os agentes em foco, bem como as potencialidades e obstáculos que permeiam o cotidiano dos profissionais das unidade de Educação Infantil no processo de promoção da alimentação adequada e saudável em contextos específicos. Espera-se que o modelo aqui proposto possa subsidiar a produção científica sobre promoção da alimentação adequada e saudável na escola, e inspire o desenvolvimento de práticas e políticas públicas de promoção da alimentação adequada e saudável nesse âmbito.
